# Interleukin inhibitors and the associated risk of candidiasis

**DOI:** 10.3389/fimmu.2024.1372693

**Published:** 2024-03-28

**Authors:** Sabir Khan, Hazrat Bilal, Muhammad Nadeem Khan, Wenjie Fang, Wenqiang Chang, Bin Yin, Ning-jing Song, Zhongrong Liu, Dongxing Zhang, Fen Yao, Xun Wang, Qian Wang, Lin Cai, Bing Hou, Jiayue Wang, Chunyan Mao, Lingxi Liu, Yuebin Zeng

**Affiliations:** ^1^ Department of Dermatology, The Second Affiliated Hospital of Shantou University Medical College, Shantou, Guangdong, China; ^2^ Department of Microbiology, Faculty of Biological Sciences, Quaid-I-Azam University, Islamabad, Pakistan; ^3^ Department of Dermatology, Changzheng Hospital, Second Military Medical University, Shanghai, China; ^4^ School of Pharmacy, Shandong University, Qingdao, Shandong, China; ^5^ Department of Dermatovenereology, Chengdu Second People’s Hospital, Chengdu, China; ^6^ Department of Dermatology, Tongren Hospital, Shanghai Jiao Tong University, School of Medicine, Shanghai, China; ^7^ Department of Dermatology, First Affiliated Hospital of Guangzhou Medical University, Guangzhou, Guangdong, China; ^8^ Department of Dermatology, Meizhou Dongshan Hospital, Meizhou, Guangdong, China; ^9^ Department of Dermatology, Meizhou People’s Hospital, Meizhou, Guangdong, China; ^10^ Department of Pharmacy, Shantou University School Medical College, Shantou, China; ^11^ Department of Clinical Laboratory, Skin and Venereal Diseases Prevention and Control Hospital of Shantou City, Shantou, Guangdong, China; ^12^ Department of Dermatology, West China Second University Hospital, Sichuan University, Chengdu, Sichuan, China

**Keywords:** interleukins (ILs), immune system, signaling pathways, interleukin inhibitors, candidiasis

## Abstract

Interleukins (ILs) are vital in regulating the immune system, enabling to combat fungal diseases like candidiasis effectively. Their inhibition may cause enhanced susceptibility to infection. IL inhibitors have been employed to control autoimmune diseases and inhibitors of IL-17 and IL-23, for example, have been associated with an elevated risk of *Candida* infection. Thus, applying IL inhibitors might impact an individual’s susceptibility to *Candida* infections. Variations in the severity of *Candida* infections have been observed between individuals with different IL inhibitors, necessitating careful consideration of their specific risk profiles. IL-1 inhibitors (anakinra, canakinumab, and rilonacept), IL-2 inhibitors (daclizumab, and basiliximab), and IL-4 inhibitors (dupilumab) have rarely been associated with *Candida* infection. In contrast, tocilizumab, an inhibitor of IL-6, has demonstrated an elevated risk in the context of coronavirus disease 2019 (COVID-19) treatment, as evidenced by a 6.9% prevalence of candidemia among patients using the drug. Furthermore, the incidence of *Candida* infections appeared to be higher in patients exposed to IL-17 inhibitors than in those exposed to IL-23 inhibitors. Therefore, healthcare practitioners must maintain awareness of the risk of candidiasis associated with using of IL inhibitors before prescribing them. Future prospective studies need to exhaustively investigate candidiasis and its associated risk factors in patients receiving IL inhibitors. Implementing enduring surveillance methods is crucial to ensure IL inhibitors safe and efficient utilization of in clinical settings.

## 1 Introduction

Candidiasis is one of the most common fungal infections affecting humans, caused by *Candida* species (1). These opportunistic pathogens are typically present on the skin and mucous membranes of healthy individual’s without causing infections (2). However, the risk factors of candidiasis are heightened in patients with compromised immune systems, often due to conditions such as diabetes, renal failure or liver failure (1, 3).

**Figure 1:**
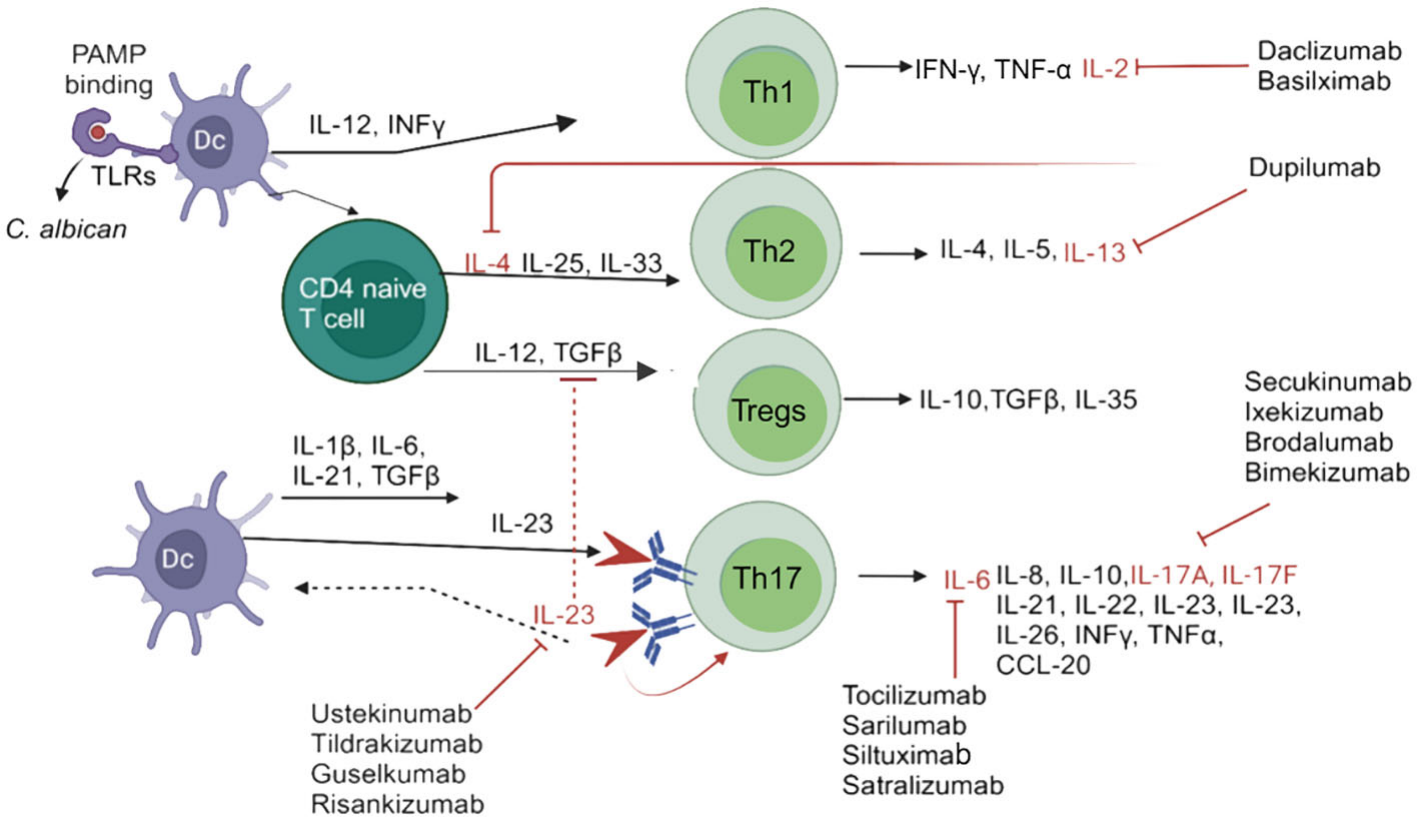
Schematic diagram of *candidiasis* and the immune system’s response for eliminating Candida species. In response to *Candida* species, DCs are activated, which promotes CD4+T cells or Th1, Th2, Tregs, and Th17 immune cells through various cytokines, in order to activate interleukins, and block by different interleukins inhibitors. IL-4, IL-13 inhibitors=Dupilumab; IL-6 inhibitors= Tocilizumab, Sarilumab, Siltuximab, Satralizumab; IL-17A, IL-17F inhibitors= Secukinumab, Ixekizumab, Brodalumab, Bimekizumab; IL-2 i inhibitors=Daclizumab, Basilximab; IL-23 inhibitors= Ustekinumab, Tildrakizumab, Guselkumab and Risankizumab. PAMP, pathogen-associated molecular patterns; IFN-γ, Interferon-gamma; IL, interleukin; Th, T helper cells; TGFβ, transforming growth factor beta; TLRs, Toll-like receptors. TNF-α, Tumor necrosis factor alpha.

The interaction of *C. albicans* Toll-like receptors (TLRs) or C-type lectin receptors is a multifaceted phenomenon due to the potential collaboration between TLRs and other pattern recognition receptors (PRRs). Additionally, the expression of fungal ligands on the surface is contingent upon the specific strain and morphotype, i.e., yeasts or hyphae, thereby influencing the resultant immune response (Th1/Th2/Th17; **Figure 1**) (4). Furthermore, a family of signaling proteins known as interleukins (ILs) plays a crucial role in the immune system. ILs are produced by various immune cells, including T cells, B cells, macrophages, and dendritic cells (DCs) (5). INF γ, TNFα, IL-2, IL-4, IL-5, IL-6, IL-8, L-10, IL-13, IL-10, TGFβ, IL-17, IL-21, IL-22 IL-23 and IL-26 are produced by Th1/Th2/Tregs/Th17 immune cells produced at the site of infections (**Figure 1**). ILs play a significant role in the host’s defense against bacterial and fungal pathogens (6–10) (**Figure 1**).

IL inhibitors such as anakinra, canakinumab, basiliximab, daclizumab, and tocilizumab, have been used to treat IL-related autoimmune diseases. Based on current knowledge, specific IL inhibitors targeting IL-8, IL-10, IL-18, and IL-22 have not yet been reported. Cytokines also participate in host defense mechanisms against *Candida* species (11–13). Thus, certain IL inhibitors has been linked with an increased incidence of *Candida* infections (14). For instance, risankizumab, a selective anti-IL-23 antibody approved for the treatment of psoriasis, has been observed to induce mucocutaneous candidiasis in approximately 3.4% of cases (15). This interaction between candidiasis and IL inhibitors is a serious concern for patients undergoing treatment with such inhibitors (16).

Several studies have been compared the safety of the inhibitor medicines. For example, individuals with psoriasis receiving treatment in animal models, the inhibitor of tildrakizumab has shown a lower risk of candidiasis than Ustekinumab (17). In a clinical trial, a 2.3% incidence of candidiasis was observed in patients receiving Ustekinumab (17). However, information on risk factors associated with candidiasis is still scarce. This limitation may be attributed to the recency of that approval of IL inhibitors for treating autoimmune diseases, resulting in insufficient data (15).

Increasing awareness among physicians and researchers might help reduce the risk factors and occurrence of candidiasis. Therefore, this review aims to provide comprehensive insights into ILs and their role in the host immune response against candidiasis, and to discuss IL associated with candidiasis.

## 2 Candidiasis and the impact of interleukin inhibitors

### 2.1 Immune response to *Candida* infection

As specialized antigen-presenting cells (APCs), macrophages play a crucial role in the immune system’s defense against *Candida*. Upon activation by chemical signals from infected cells, they are recruited and phagocytize *C. albicans*, thereby eliminating and producing fungal protein antigens. DCs identify *Candida* species through PRRs interacting with pathogen-associated molecular patterns (PAMPs) on the fungal cell wall (18, 19). DCs facilitate the transfer of fungal antigens to lymph nodes, initiating an immune response and leading to T-cell development against *Candida* for long-term immunological defense (20, 21). DCs exhibit antigen-specific immune responses to *C. albicans*, with Langerhans cells driving Th17 responses. In contrast, cutaneous DCs without Langerhans cells stimulate Th1 and cytotoxic T-lymphocyte (CTL) responses, presenting antigens through the major histocompatibility complex class I (MHC I) route (10). T-lymphocytes recognize these antigens through receptors, releasing cytokines and differentiating into various Th cells. These dynamic interactions contribute collectively to the immune system’s ability to combat *Candida* infections (21, 22).

ILs modulate specific pathways during *Candida* infections. IL-1 and IL-6 mutually influence the production of IL-8, IL-17, and IL-22 through mitogen-activated protein kinase (MAPK) or nuclear factor-kappa B (NF-kB) specific pathways (23). IL-1 plays a role in recruiting immune cells and promoting inflammation in response to *Candida* infection. Furthermore, IL-12 regulates T cell proliferation and differentiation, with IL-2 enhancing interferon-gamma (INF-γ) production through the januskinase signal transducer and transcription factor (JAK-STAT) pathway during *Candida* infection (21, 23). IL-4 stimulates Th2 cell differentiation, antibody production, and IL-1, IL-6 and IL-8 production. IL-4 also plays a role in developing a Th2 immune response, inadvertently promoting fungal growth (24).

IL-10, inhibits inflammation and modulates the immune response, simultaneously blocking the production of IL-1, IL-6 and IL-8. IL-10 may contribute to *Candida’s* evasion strategy by reducing the inflammatory response, and increasing fungal persistence. Furthermore, IL-12 promotes the differentiation of Th1 cells and stimulates IFN-γ cells (21, 24). IL-17 comprises various subtypes, including IL-17A, IL-17B, IL-17C, IL-17D, IL-17E (also known as IL-25) and IL-17F. These ILs can activate the production of IL-6, IL-8, and IL-1β. IL-17 induces the production of IL-6 through the NF-kB pathway. During *Candida* infection, IL-17 play an important role in promoting inflammation and recruiting neutrophils (25). Conversely, IL-18 plays a pivotal role in enhancing the production of IFN-γ and modulating immune responses. It also promotes the production of IL-1 and IL-17. In the context of candidiasis, IL-18 stimulates cellular immunity and enhances IFN-γ production (26).

IL-22 plays a crucial role in promoting the function of epithelial cells and tissue repair, activating the production of IL-1, IL-6 and IL-8. During candidiasis, IL-22 enhances the immune cell response and facilitates tissue repair. IL-23 encourages Th17 cell development and induces the production of IL-17, serving as a critical factor in the host immunological response to *Candida* infection (12, 27). The primary homeostatic function of the IL-23/IL-17 pathway in the host is to defend against fungal and bacterial pathogens at mucosal barriers, concurrently overseeing barrier function (28). IL-23, a member of the IL-12-type cytokine family, comprises the p19 and p40 subunits, connected by a disulfide bond. Its interaction is mediated by two receptor chains, IL-12Rβ1 (shared with IL-12) and the IL-23-specific IL-23R (29)

The binding of IL-12 to the IL-12Rβ1 and IL-12Rβ2 receptors activates immune cells, including NK cells and T cells (30). Additionally, IL-12Rβ1 activation leads to the production of pro-inflammatory cytokines, such as IFN-γ and IL-2, promoting a Th1 response and enhancing the immune system’s ability to combat fungal pathogens (31, 32). Anomalous expression of IL-23 signaling can result in autoimmune diseases. In the context of candidiasis, IL-23 plays a crucial role in activating immune cells, including neutrophils and phagocytesthat enhance the immune response against fungal pathogens at the site of infection and facilitate tissue repair (10). The lack of IL-23 signaling pathways has been linked to increased susceptibility to candidiasis, underscoring the critical role of IL-23 in host defense against *Candida* (33). Immune cells also maintain a homeostasis of defense immunity to prevent excessive immune responses triggered by autoimmune diseases such as psoriasis, RA, and MS (34, 35). However, while IL inhibitors therapy has been used to treat autoimmune diseases, it comes with the risk of candidiasis, a significant concern during treatment.

### 2.2 Host mechanism by which interleukin inhibitors increase the risk of candidiasis

*Candida* species exhibit the capacity to infiltrate cells at the site of infection by binding to specific receptors. Initially, immune cells, such as macrophages and DCs, recognize molecules on the surface of *Candida* (10). The recognition of *C. albicans* manam by APS leads to the production of interleukins including IL-23 and chemokines (36). Th17 cells play a vital role by secreting various cytokines, notably IL-6, IL-17, IL-22, and IL-23, crucial for immune protection against *Candida* at mucosal sites throughout the body (25, 37). These cytokines also in attract and activate neutrophils, which engulf *Candida* species. Phagocytes, such as immune cells, respond by releasing antifungal proteins (e.g., Chemokine (C-C motif) ligand 2 (CCL2), antimicrobial peptides, and β-defensin) and pro-inflammatory chemokines (38, 39).

The described response mechanism effectively eliminates infectious molecules or kills *Candida* species within cells. However, neutralizing specific ILs, such as IL-17 RA (IL-17RA-/-) or IL-23p19 (IL-23p19-/-), has been found to increase susceptibility to oropharyngeal candidiasis and systemic candidiasis (25, 38). IL inhibitors are used to treat autoimmune diseases by inhibiting the recruitment and activation of immune cells such as DCs, macrophages, and neutrophils (40). Consequently, this inhibition hampers the production of chemokines and antimicrobial peptides increasing the risk of candidiasis due to the blockage of immune cell responses, allowing *Candida* to breach the epithelial barrier (41, 42). However, numerous studies have found that most candidiasis cases resulting from IL inhibitors are mild to moderate and do not lead to systemic infection (43).

## 3 IL inhibitors and *Candida* infection in immune mediated diseases

### 3.1 IL-1 inhibitors: anakinra, canakinumab, and rilonacept

IL-1 inhibitors, including anakinra, canakinumab, and rilonacept, are used to counteract the effects of IL-1, a cytokine that promotes inflammation and controls immune responses and inflammation. These inhibitors prevent the action of IL-1 and its receptors to reduce inflammation (44, 45). For instance, anakinra binds to IL-1 receptors, thereby blocking their binding with IL-1. This agent effectively manages joint pain and inflammation and is commonly used to treat rheumatoid arthritis (RA) (46).

Canakinumab is a monoclonal antibody specifically designed to target IL-1, inhibiting both its activity and downstream signaling. IL-1 activity is believed to play a role in the pathophysiology of diseases, such as cryopyrin-associated periodic syndromes (CAPS) and systemic juvenile idiopathic arthritis (SJIA). On the other hand, rilonacept, is a fusion protein consisting of an Fc region and the extracellular portion of the IL-1 receptor. It binds to both IL-1α and IL-1β, preventing their signaling by interacting with IL-1 receptors. Rilonacept is used to treat condition like CAPS and familial mediterranean fever (FMF) (47). In a randomized, double-blind trial phase with placebo followed by open-label extension for RA. 1346 patients with RA received anakinra subcutaneous injection for three years. Although, *candidal* esophagitis was reported in one patient approximately 2.5 years after initiating anakinra (100 mg/day) treatment (**Table 1**) (48).

**Table 1:** Interleukin inhibitors, their targets, and the associated risk of candidiasis.

Biologics	Targets	Diseases	Candidiasis	References
Anakinra	IL-1	rheumatoid arthritis	Candidal esophagitis	(48)
Daclizumab	IL-2	multiple sclerosis	Oral candidiasis, Vulvovaginal candidiasis	(49)
Sarilumab	IL-6	rheumatoid arthritis	Candidal bronchitis	(50)
Tocilizumab	IL-6	rheumatoid arthritis, systemic juvenile idiopathic, arthritis, cytokine release syndrome	Candidemia	(51–53)
Brodalumab	IL-17A	plaque psoriasis, psoriasis	Mucocutaneous candidiasis	(54–56)
Secukinumab	IL-17A	psoriasis, ankylosing spondylitis	Candidiasis	(57, 58)
Ixekizumab	IL-17A	psoriatic arthritis, ankylosing spondylitis	Candidiasis	(59–62)
Bimekizumab	IL-17A and IL-17F	psoriasis,	Candidiasis	(63, 64)
Ustekinumab	IL-12, IL-23	plaque psoriasis, psoriasis, psoriatic arthritis, crohn’s disease, ulcerative colitis	Mucocutaneous candidiasis, Oesophageal candidiasis	(54, 65)
Guselkumab	IL-23	plaque psoriasis, psoriasis, psoriatic arthritis	Candidiasis	(58, 66)
Risankizumab	IL-23	plaque psoriasis, psoriasis, ankylosing spondylitis, crohn’s disease	Candidiasis	(15, 67)
Tildrakizumab	IL-23	plaque psoriasis, psoriasis	Candidiasis	(68)

### 3.2 IL-2-inhibitors: daclizumab, and basilximab

Daclizumab is a monoclonal antibody specifically designed to target the IL-2 receptor (IL-2R) for treating of multiple sclerosis (MS). It selectively binds to the CD25 component of IL-2R, expressed on activated T cells. By targeting and blocking IL-2R, daclizumab effectively modulates the immune response and reduces the activity of immune cells responsible for the inflammation and damage commonly observed in MS (69, 70).

Basiliximab, a monoclonal antibody, is an immunosuppressive organ transplantation agent designed to prevent rejection. By targeting CD25, basiliximab effectively decreases the risk of organ rejection by inhibiting the activation and proliferation of T cells pivotal in immuneresponses (71). In multicenter, randomized, double-blind studies investigating MS. 2236 patients received daclizumab 150 mg or 300 mg and the first incident of infection occurred at 162 days. Patients received daclizumab 150 mg the incidence of 12 oral candidiasis, and 12 vulvovaginal candidiasis occurred (49).

### 3.3 IL-4 and IL-13, inhibitor: dupilumab

The monoclonal antibody dupilumab has been developed to target the IL-4 receptor alpha (IL-4R), effectively blocking the signaling of both IL-4 and IL-13. Dupilumab is typically used to treat allergic conditions such as atopic dermatitis and asthma (72). Recently, in a case report, patient received dupilumab (600 mg) for the treatment of severe asthma and atopic dermatitis, after the 6 week the incident of *candida* infection occurred (72).

### 3.4 IL-6 inhibitors: tocilizumab, sarilumab, siltuximab, and satralizumab

The monoclonal antibody tocilizumab targets the IL-6 receptor (IL-6R) and is used to treat various autoimmune conditions, including giant cell arteritis, cytokine release SJIA, and RA. Tocilizumab works by inhibiting IL-6 signaling by binding to both the soluble and membrane-bound forms of IL-6R (73). By effectively blocking IL-6 signaling, tocilizumab serves to decrease inflammation and manages the signs and symptoms related of autoimmune disorders (74). In a report of cumulative safety data from five core phase III trials, two ongoing extension trials, and one clinical pharmacology. Overall, 22 opportunistic infections, of which six cases were candidiasis occurred in patients treated with tocilizumab 8mg/kg for RA (75). Sarilumab is a monoclonal antibody designed to target IL-6R and used to treat moderate-to-severe active RA. Through its binding to IL-6, sarilumab effectively inhibits IL-6 signaling. IL-6 is known for its role in promoting inflammation and is involved in the pathogenesis of RA. Sarilumab reduces inflammation and alleviates the symptoms associated with rheumatoid arthritis by suppressing IL-6 signaling (76). In a study called MOBILITY, double blind, randomized, placebo-controlled phase III trial, 200 mg of sarilumab was administered subcutaneously every two weeks in combination with methotrexate for the treatment of 45 patients with active RA, and one instance of *candidal* brochitis was reported (50).

IL-6, a pro-inflammatory cytokine with implications for immunological responses and inflammation, is the target of the monoclonal antibody siltuximab. This biologic agent is primarily treats multicentric castleman disease (MCD), a rare disorder characterized by abnormal lymph node enlargement and systemic inflammation. By selectively binding to IL-6 and inhibiting it signaling, siltuximab effectively reduces the inflammatory response associated with MCD. Siltuximab helps manage the symptoms and consequences of the illness by blocking IL-6 (77).

Satralizumab, a monoclonal antibody targeting IL-6R, is utilized in the treating of neuromyelitis optica spectrum disorder (NMOSD), a rare autoimmune disorder that primarily affecting the spinal cord and optic nerves. This therapeutic agent functions by binding to IL-6R, thereby preventing the binding of IL-6 to the receptor and its activation. By impeding IL-6 signaling, satralizumab helps reduce inflammation and prevents relapses in NMOSD (78).

### 3.5 IL-17 inhibitors: secukinumab, ixekizumab, brodalumab, and bimekizumab

The monoclonal antibody secukinumab is specifically designed to selectively target and suppress the pro-inflammatory cytokine IL-17A, which play a pivotal role in autoimmune diseases, such as psoriasis, ankylosing spondylitis, and psoriatic arthritis (57). To treat ankylosing spondylitis, a dosage of secukinumab has been selectively adjusted to a range of 150 to 300 mg. However, this adjustment has been associated with candidiasis in 11 (2.3%) cases for the lower dosage and 22 (4.7%) cases for the higher dosage (57).

The humanized monoclonal antibody ixekizumab is designed to target and suppress the production of IL-17A, a cytokine associated with autoimmune conditions such as psoriasis and psoriatic arthritis (59). Notably, pivotal phase III randomized, double-blind, placebo-controlled trials for psoriasis, known as UNCOVER-1, -2, and -3, that reported a total of 2,570 patients received ixekizumab, of which first dose was 160 mg followed by 80 mg every 2 or 4 weeks. Hence,16 incidences of candidiasis were reported and interestingly, an equal number instances were observed between weeks 0 and 60 and between weeks 0 and 12 (60–62).

Next, the human IgG2 monoclonal antibody known as brodalumab targets IL-17 receptor A (IL-17RA), a protein involved in the signaling cascade of IL-17 cytokines (79). Brodalumab reduces the inflammatory response associated with autoimmune diseases like psoriasis by inhibiting IL-17RA (80). In phase III clinical studies, namely AMAGINE -1, -2, and -3, 1338 adults with significant psoriasis were randomized to receive either brodalumab 210 mg or 140 mg. In all three studies involving brodalumab, 33 candidiasis cases were reported almost of them with a higher dosage of 210 mg (54–56).

The humanized IgG1 monoclonal antibody bimekizumab is designed to selectively target and suppress the pro-inflammatory cytokines IL-17A and IL-17F, both associated with autoimmune disorders (81). Bimekizumab is often administered to individual with psoriasis. Adult patients with moderate-to-severe psoriasis participated in three active-comparator-controlled, randomized phase III clinical trial: BE SURE, BE RADIANT, and BE VIVID. These studies utilized adalimumab, secukinumab, or Ustekinumab as active comparators, respectively. Candidiasis incidence was observed in the active-comparator group (0% to 5%), while in the bimekizumab group exhibited more candidiasis cases (9% to 21%) (63, 64, 82).

### 3.6 IL-23 inhibitors: ustekinumab, tildrakizumab, guselkumab and risankizumab

IL-23 inhibitors target and block IL-23, a cytokine that plays a crucial role upstream of Th17 lymphocyte activation. Four IL-23 inhibitors, namely ustekinumab, tildrakizumab, guselkumab, and risankizumab, have received approval for the treatment of psoriasis and psoriatic arthritis (83, 84). Ustekinumab, is a monoclonal antibody (IgG1 kappa) that binds to both the p19 and p40 subunits of the IL-23 protein. This biologic effectively suppresses IL-23 and IL-12 by interacting with the p40 subunit on IL-12 (85, 86). Ustekinumab is employed in treating moderate-to-severe plaque psoriasis, psoriatic arthritis, Crohn’s disease, and ulcerative colitis (87, 88).

The efficacy of ustekinumab and the occurrence of candidiasis infections have been studied in various clinical trials. In the UNITI trials, two cases of oesophageal candidiasis were reported: one in the placebo group and one in the group receiving 90 mg of ustekinumab every eight weeks (65). In another study encompassing two phases (AMAGINE-2 and AMAGINE-3) and in the CLEAR trials, brodalumab (210 mg or 140 mg) was compared with ustekinumab (45 mg or 90 mg) or placebo, a total of 3712 patients were examined and mucocutaneous *Candida* infections were reported in 1.3% of patients treated with ustekinumab at week 52 (54).

**Figure 2:**
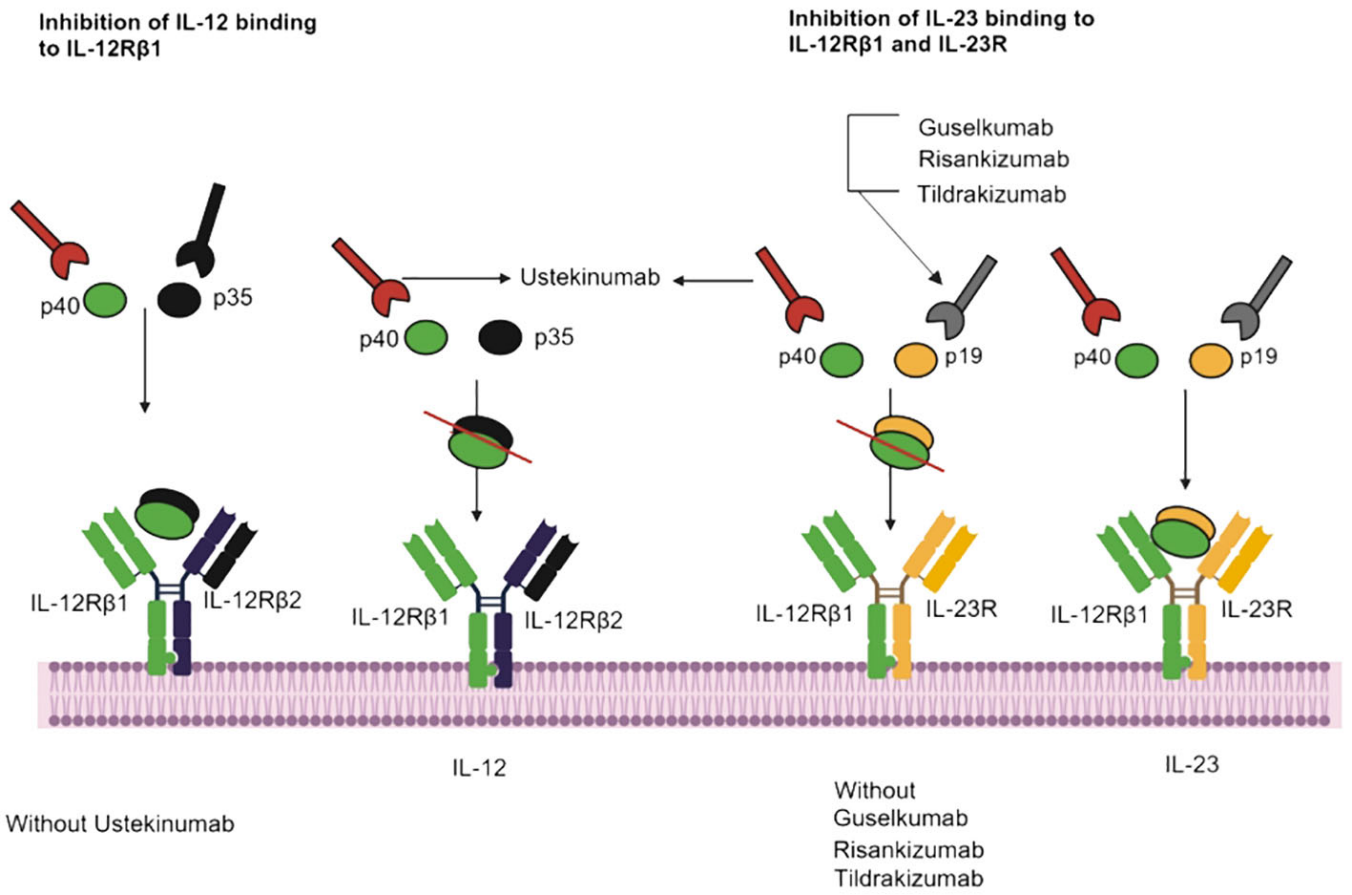
IL-23 inhibitors, binding sites, and mechanisms of action. Ustekinumab interacts with IL-12Rβ1, and Guselkumab, Risankizumab and Tildrakizumab inhibit IL-23 and IL-12Rβ1.

Tildrakizumab is a monoclonal antibody of the IgG1 class that specifically targets the p19 subunit of IL-23, a cytokine involved in the inflammatory responses of the immune system and implicated in conditions like psoriasis and psoriatic arthritis (**Figure 2**) (89, 90). Consequently, tildrakizumab effectively treats autoimmune diseases by inhibiting IL-23 and reducing inflammation (91). A comprehensive efficacy and safety trial, encompassed 391 patients with active psoriatic arthritis who received various doses of tildrakizumab (200 mg every four weeks, 100 mg every 12 weeks, or 20 mg every 12 weeks), alongside a placebo. In this trial, cases of candidiasis minimal (0.5% of all patients). Interestingly, both cases of candidiasis were observed in patients receiving tildrakizumab at a dosage of 200 mg every 4 weeks (68). Furthermore, guselkumab is an IgG1λ monoclonal antibody that inhibits IL-23 by targeting the p19 subunit (92). This biologic is used in the treatment of various conditions like pustular psoriasis, psoriatic arthritis, and chronic plaque psoriasis (93). In seven phase II/III studies (X-PLORE, VOYAGE 1, VOYAGE 2, NAVIGATE, ORION, ECLIPSE, Japan registration, randomized, double-blind, placebo-controlled trials for psoriasis, the 4252 patients who received guselkumab at dosage of 50-100 mg for weeks 0-16 weeks, three patients developed *Candida* infections, including two cases of vulvovaginal candidiasis and one of oral candidiasis. In the guselkumab (100 mg) treatment group, two patients developed candidiasis, comprising one case of vulvovaginal candidiasis and one of skin *Candida* (66). In the above seven phase II/III studies, 2891 patients were treated for psoriasis with guselkumab for up to 5 years. They suffered *Candida* infections, including vulvovaginal candidiasis (*n*=23), skin *Candida* (*n*=15), oral candidiasis (*n*=9), *Candida* infection (*n*=2), genital c andidiasis (*n*=2), and balanitis *Candida* (*n*=1) (66).

Risankizumab, a monoclonal antibody, has obtained approval for treating specific inflammatory conditions, such as moderate-to-severe psoriasis and psoriatic arthritis (94, 95). During placebo-controlled trials, open-label risankizumab clinical trials, 1306 patients were treated with 150 mg of risankizumab for primary psoriasis over a 16-week period, two cases of *Candida* infection occurred in this cohort (67). The safety and risk profiles of various IL inhibitors have been compared to assess their efficacy in addressing psoriasis. Notably, in patients treated with risankizumab, the reported rate of serious infection stood at 1.0 to 1.6 per 100 patient-years, compared to rates of 1.1 in the placebo group, 2.1 in patients treated with adalimumab and 5.3 in those treated with ustekinumab. Mucocutaneous candidiasis was noted in 3.4% of cases (15).

Additionally, in a phase 3 randomized controlled trial comparing guselkumab to secukinumab for the treatment of moderate-to-severe psoriasis, *Candida* infections occurred in 2% of the guselkumab-treated group and in 6% of those who received secukinumab (58). A recent trial investigating the new IL-17 inhibitor bimekizumab reported oropharyngeal candidiasis in 15% of patients, compared to 1% in those treated with ustekinumab (64). Patients receiving brodalumab were administered 210 mg or 140 mg maintenance doses every 2, 4, or 8 weeks between weeks 12 and 52. In the induction phase, patients in the placebo group received 210 mg of brodalumab every 2 weeks, while those in the ustekinumab group continued their treatment every 12 weeks. The incidence of mucocutaneous candidiasis was 4.8% among patients receiving brodalumab and 1.3% among those receiving ustekinumab at week 52 (54).

Overall, the risk of candidiasis associated with using IL12/23 inhibitors appear to be lower than that observed with IL17 inhibitors. Therefore, anti-IL-12/23 agents may be considered preferable for the treatment of autoimmune diseases. The variability in risk profile emphasized the important of selecting and tailoring treatment approaches, necessitating a thorough evaluation of the individual characteristics and factors associated with different groups of biological drugs. This approach ensures a comprehensive evaluation of individual patient factors, contributing to more inform and specialized medical treatment options.

### 3.7 Real world data on the risk of candidiasis during IL inhibitors

Several studies have examined the use of IL inhibitor therapy and its association with candida infection (**Table 1**). From June to November of 2021, 40 eligible patients received IL-13 and only had experienced candidiasis (96). Also, a single patient recently received dupilumab for the treatment of severe asthma and atopic dermatitis and an incident of *Candida* infection occurred. A total of 303 patients received IL-6 inhibitors and 12 had *Candida* infection including five candidemia with tocilizumab (400 mg) after two weeks (51, 52, 97, 98).

Furthermore, between 2017 and 2018, research was conducted where 391 patients received IL-23 inhibitors, with only two patients (0.5%) experiencing *Candida* infections both were on tildrakizumab 200 mg at four weeks (68). In 2021, a total of 955 patients received risankizumab at a dosage of 150 mg for up to 208 weeks, resulting in 20 reported cases of candidiasis. These cases manifested as seven cases of oral candidiasis, seven of genital candidiasis, four of esophageal candidiasis, one of intestinal candidiasis, and one of skin candidiasis (67). To further elucidate the study, a comprehensive analysis was conducted using aworld health organization (WHO) database containing 16,343,451 individual case safety reports. Among the reports, 50,353 cases pertained to candidiasis, of which 33,735 were categorized as proven or probable. Among the candidiasis cases, 427 reports (356, 1.05%, proven/probable candidiasis) were linked to anti-IL-17 drugs as the suspected cause, while 88 reports (63, 0.18%, proven/probable) were associated with anti-IL12/23 drugs (99). A population-based study examined the incidence of infections in patients with psoriasis with interleukin (IL)-23 inhibitors (IL-23i) and IL-17 inhibitors (IL-17i). A 5272 of patients received IL-23, IL-23i inhibitor, after which 104 cases of mucocutaneous candidiasis were noticed. In the same case, 15,160 patients received an IL-17 inhibitor, and mucocutaneous candidiasis was reported in 560 patients (100). In a retrospective study, 4866 adult patients with psoriasis were treated with an interleukin IL-17 or IL-23 inhibitor between 1 February 2015 and 31 October 2021. Due to the use of IL-17 inhibitor, 102 patients were infected with *candida*, while only 8 contracted the infection due to IL-23 inhibitor (101).

Moreover, a review compiled the clinical trial and real-world studies related to candidiasis incidences using IL-17 inhibitors. Here, the different IL-17 inhibitors and associated risk factors of candidiasis were compared. Additionally, guideline and safety assessment were provided in detail to healthcare professionals before prescribing IL-17 inhibitors (102). Moreover, a study examining biologics, including ustekinumab, and their potential for candidiasis risk, collected data from WHO VigiBase, the Population-based Drug Prescriptions Registry (PHARMO), and the Netherlands Psoriasis Cohort. This study revealed that of the 17,398 patients exposed to ustekinumab, 63 (0.36%) developed candidiasis. The manifestation included mucocutaneous (n=37), cutaneous (n=19), oropharyngeal (n=12), vulvovaginal (n=10), esophageal (n=6), onychomycosis (n=4), and candidemia (n=1) (99).

## 4 IL inhibitors and *Candida* infection in Covid-19

According to the World Health Organisation (WHO), an estimated15 million individuals died in the years of 2020- 2021 due to the COVID-19 pandemic (103). However, there were several interleukin inhibitors used for the treament of COVID-19 diseases (104). During phase 2a of a randomized, double-blind, placebo-controlled trial investigating treatment for COVID-19, 40 patients with Coronavirus disease received dupilumab given as two 300 mg subcutaneous injections at day 0 and with additional maintenance doses of 300 mg or placebo given on days 14 and 28. One patient who incident of oral candidiasis was reported during the whole study (96).

Recently, a study reported that out of 43 patients with severe COVID-19 pneumonia treated with tocilizumab, three (6.9%) developed candidemia (**Table 1**) (51). Another study compared tocilizumab use in patients with candidemia both with and without COVID-19 and found that it was 30 times more prevalent in COVID-19-associated candidemia (18.8% vs. 0.5% without COVID-19; p < 0.0001) (53). In a retrospective, observational cohort study, 179 patients with severe COVID-19 pneumonia patients were treated with tocilizumab (8 mg/kg body weight) twice a day, of which two had candidemia within 12 days (52). In another retrospective cohort study, tocilizumab (400 mg) was given to 65 patients for the treatment of severe COVID-19, and one patient had candidemia day 18 (97). In another example of an observational cohort study, 16 patient with COVID-19 were administered with tocilizumab (8 mg/dose) and five patient had candidemia (98). Furthermore, in an open-label therapy, a combination of tocilizumab (8 mg/kg up to 800 mg) and anakinra (100–300 mg) were given to thirty-one patients with severe COVID-19 pneumonia, in which one patient infected with candidal infection (105).

## 5 Discussion and conclusion

This comprehensive review highlights the intricate relationships between IL inhibitors, immunological responses, and the associated risks of opportunistic infections, such as candidiasis. Clinicians must possess a profound understanding of the mechanisms of these inhibitors to make informed clinical decisions and strike an appropriate balance between suppressing the immune system and preventing potential adverse effects. The positive impact of targeted therapy is most evident with IL-1 inhibitors, which reduce inflammation by blocking IL-1 signaling (106). IL-2 inhibitors provide valuable insight into precise immune regulation by targeting activated T cells (107). Most importantly, instances of *Candida* infections associated with IL-1 and IL-2 inhibitors are rarely reported. Furthermore, IL-4 and IL-13 inhibitors are promising for allergy treatment, albeit with candidiasis concerns, and IL-6 inhibitors curb autoimmune responses by interrupting IL-6 signaling interleukin (108). Notably, IL-17 inhibitors, used intreating complex autoimmune diseases, represent significant therapeutic options. Furthermore, IL-23 inhibitors block IL-23, a cytokine upstream of and crucial for the activation of Th17 lymphocytes consequently reducing the production of IL-17, IL-12, and IL-22 (109). However, limitations to the therapeutic potential of IL inhibitors do exist. This review underscores the risks of candidiasis associated with IL inhibitors several classes. Professionals must navigate these complex scenarios as the field evolves, weighing the benefits of immune system regulation against potential infection risks. In this dynamic setting, it is crucial for healthcare professional from various fields including, rheumatologists, immunologists, infectious disease experts, and other medical specialists, to collaborate toward the effective use of IL inhibitors.

In conclusion, this review comprehensive describes the intricate interplay between IL inhibitors, immunological responses, and the looming threat of candidiasis. *Candida* infections commonly occur hospitalized patients, particularly in immunocompromised. Hence, the use of IL inhibitor in the treatment of autoimmune diseases or COVID-19 poses a serious concern, as they inhibits immune response, and increasing the risk of candidiasis. It is crucial for clinicians to carefully balance therapeutic advantages with the risk of opportunistic infections as biological treatments reshape the management of autoimmune diseases. The advent of precision medicine heralds a new era of focused treatments, where a deep understanding of the complex functions of the immune system will inform individual choices.

## Author contributions

SK: Conceptualization, Methodology, Writing – original draft, Writing – review & editing. HB: Formal analysis, Methodology, Writing – review & editing. MK: Formal analysis, Investigation, Writing – review & editing. WF: Validation, Visualization, Writing – review & editing. WC: Formal analysis, Writing – review & editing. BY: Formal analysis, Writing – review & editing. N-JS: Formal analysis, Writing – review & editing. ZL: Formal analysis, Writing – review & editing. DZ: Methodology, Writing – review & editing. FY: Writing – review & editing. XW: Investigation, Writing – review & editing. QW: Investigation, Writing – review & editing. LC: Project administration, Resources, Writing – review & editing. BH: Validation, Visualization, Writing – review & editing. JW: Data curation, Writing – review & editing. CM: Validation, Writing – review & editing. LL: Data curation, Conceptualization, Formal analysis, Funding acquisition, Investigation, Methodology, Project administration, Resources, Software, Supervision, Validation, Visualization, Writing – review & editing. ZY: Investigation, Methodology, Project administration, Resources, Writing – review & editing.
